# Association between peak D-dimer levels within 24 hours of admission and the risk of infected pancreatic necrosis in hyperlipidemic acute pancreatitis: a retrospective analysis

**DOI:** 10.3389/fmed.2026.1903091

**Published:** 2026-08-25

**Authors:** Xuexiao Chen, Lidan Su, Daguan Zhang, Aidi Chen, Xiaoyan Liu

**Affiliations:** Department of Gastroenterology, The First Affiliated Hospital of Wenzhou Medical University, Wenzhou, China

**Keywords:** D-dimer, hyperlipidemic acute pancreatitis, infected pancreatic necrosis, nomogram, predictive model

## Abstract

**Objective:**

The goal of this study is to analyze the relationship between the peak D-dimer levels measured within 24 h of admission and the risk of infected pancreatic necrosis (IPN) in patients with hyperlipidemic acute pancreatitis (HLAP) and to create and validate an early in-hospital risk stratification model of IPN.

**Methods:**

The study was conducted as a retrospective study on 286 HLAP patients from January 2023 – June 2025, divided into IPN (*n* = 47) and non-IPN (*n* = 239) groups. A separate time-validation group was collected (*n* = 62) from July 2025 to December 2025. Multivariate logistic regression was used to identify independent risk factors for IPN and build the nomogram. The ROC curve, calibration curve, Hosmer-Lemeshow test, Delong test, and DCA were performed to evaluate model performance. Using restricted cubic spline (RCS), the dose–response relationship between D-dimer and IPN risk was evaluated.

**Results:**

D-dimer, procalcitonin (PCT), MCTSI score, and persistent organ failure (POF) were independent risk factors for IPN (all *p* < 0.05). RCS analysis revealed a linear dose–response relationship between D-dimer and IPN risk (P for overall <0.001, P for nonlinear = 0.119). The combined model incorporating D-dimer, PCT, MCTSI, and POF achieved an AUC of 0.82 (95% CI: 0.75–0.89), with sensitivity of 0.70, specificity of 0.82, and accuracy of 0.80. Calibration was good (Hosmer-Lemeshow *p* = 0.806), and DCA demonstrated clinical net benefit across threshold probabilities of 0.1–0.7. In the temporal validation cohort, the model yielded an AUC of 0.73 (95% CI: 0.56–0.89), with no significant difference from the derivation cohort (*p* = 0.319). Given the limited sample size of the validation cohort and its single-center origin, these findings should be considered preliminary.

**Conclusion:**

Elevated peak D-dimer level within 24 h of admission is independently associated with an increased risk of IPN in HLAP patients in a linear positive manner. The early in-hospital risk stratification model comprised of D-dimer, PCT, MCTSI and POF demonstrates additionally good predictive performance, clinical utility and risk stratification during the early course of hospitalization for personalized treatment decision.

## Introduction

The incidence of hyperlipidemic acute pancreatitis (HLAP) has been rising year by year and has become one of the leading causes of acute pancreatitis (1, 2). Compared with acute pancreatitis caused by other etiologies such as biliary tract diseases, patients with HLAP have the clinical characteristics of younger age at onset, more severe condition, and higher incidence of complications (3). Infectious pancreatic necrosis (IPN) is one of the most serious local complications of HLAP (4). The occurrence of IPN often leads to prolonged disease course, significant increase in medical costs, and even some studies have shown that its mortality rate can reach 15–35% (5, 6). Despite recent advances in intensive care and minimally invasive debridement techniques, the early identification of IPN remains a challenge. Since IPN is typically diagnosed at a late stage of the disease, by which time the optimal window for intervention has already been missed (7, 8), screening for reliable and simple biomarkers early in hospitalization to predict the risk of IPN is of significant clinical importance for optimizing treatment strategies and improving patient outcomes.

D-dimer is a specific degradation product of cross-linked fibrin and is routinely used to rule out thrombotic disorders (9). Increasing evidence has also demonstrated its potential value in assessing the severity and prognosis of acute pancreatitis (10, 11). However, acute pancreatitis is a clinically heterogeneous disease, and its underlying etiologies differ substantially in pathophysiological mechanisms. Compared with biliary or other forms of acute pancreatitis, HLAP is characterized by severe hypertriglyceridemia, excessive free fatty acid release, endothelial injury, activation of the coagulation cascade, and pancreatic microcirculatory dysfunction (12). These pathological processes are closely linked to fibrin formation and degradation and therefore may directly influence circulating D-dimer levels. Previous studies have largely evaluated D-dimer in mixed acute pancreatitis populations or focused primarily on disease severity, organ failure, or mortality, whereas evidence regarding its association with infected pancreatic necrosis in a homogeneous HLAP population remains limited (13, 14). To reduce etiological heterogeneity and better characterize the disease-specific relationship between coagulation activation and IPN development, the present study deliberately focused on patients with HLAP.

Based on the above background, this study intends to conduct a retrospective analysis of the clinical data of HLAP patients to explore the association between the D-dimer level within 24 h of admission and the risk of IPN occurrence. At the same time, it will assess the predictive value of other potential risk factors. By constructing multivariate regression models and developing early in-hospital risk stratification model, this study aims to identify independent risk factors associated with IPN and provide an evidence-based foundation for the early identification of patients at high risk for IPN among those with HLAP, and to offer guidance for clinical decision-making and the development of personalized treatment plans.

## Materials and methods

### Research design and patient population

This study is a single-center retrospective observational study, and the data are derived from The First Affiliated Hospital of Wenzhou Medical University. By searching the hospital’s electronic medical record system, the data of patients diagnosed with HLAP from January 2023 to June 2025 were collected. Consecutive sampling was employed, and all eligible patients admitted during the study period were screened in chronological order according to the predefined inclusion and exclusion criteria. A total of 313 patients were initially identified from the electronic medical record system. After applying the exclusion criteria, 27 patients were excluded from the final analysis: 5 patients were transferred from other institutions with a documented diagnosis of IPN prior to admission, 10 patients lacked contrast-enhanced CT data within the required 72–96 h window due to renal impairment (*n* = 7) or iodine allergy (*n* = 3) and had no alternative imaging available for MCTSI scoring, 7 patients had incomplete key laboratory data (missing D-dimer or PCT measurements within 24 h of admission), 3 patients had coexisting pancreatic malignancies, and 2 patients had a history of severe hematological disorders requiring long-term immunosuppressive therapy. Ultimately, a total of 286 patients were included in the primary analysis cohort. Furthermore, to verify the stability and generalization ability of the prediction model, using exactly the same inclusion and exclusion criteria, an additional 62 patients with HLAP who were admitted from July 2025 to December 2025 were collected as an independent clinical validation cohort. Our institution is a large tertiary referral center in southern Zhejiang Province, comprising three hospital campuses and serving a catchment population of approximately 30 million. This high-volume setting, combined with the increasing proportion of HLAP among acute pancreatitis etiologies nationwide, accounts for the relatively large number of HLAP cases included in this study. This study complies with the ethical principles of the Declaration of Helsinki, and the study protocol has been approved by the Ethics Committee of The First Affiliated Hospital of Wenzhou Medical University.

### Sample size considerations

This was a retrospective cohort study, and no formal sample size calculation was performed before study initiation. All consecutive eligible patients admitted during the predefined study period were included to minimize selection bias and reflect routine clinical practice. During model development, the number of outcome events relative to the number of candidate predictors was considered as a descriptive measure of model complexity. The primary prediction model included four predictors, corresponding to an events-per-variable (EPV) of approximately 11.75 (15). We acknowledge that the fully adjusted analyses additionally included age, sex, and body mass index, resulting in a lower effective EPV. Therefore, the EPV is reported as a descriptive reference rather than the sole justification for sample size adequacy, consistent with contemporary recommendations for clinical prediction model development.

### Inclusion and exclusion criteria

Inclusion criteria: (1) Age ≥ 18 years old; (2) Patients who meet the 2012 revised Atlanta Classification AP diagnostic criteria, that is, possess at least two of the following three items: acute onset of upper abdominal pain, serum amylase and/or lipase concentration ≥ three times the upper limit of normal, and abdominal imaging examination showing characteristic changes of AP (16); (3) Meets the diagnostic criteria of HLAP: Serum triglycerides ≥11.30 mmol/L, or serum triglycerides are 5.65–11.30 mmol/L and the serum appears creamy, while other causes are excluded (17); and (4) Patients with complete clinical data.

Exclusion criteria: (1) Patients referred from other institutions with a documented diagnosis of IPN established before the index admission to our hospital; (2) Merging pancreatic tumors; (3) Pregnant or lactating women; and (4) Having a history of severe blood disorders or long-term use of immunosuppressants.

All patients included in this study were admitted to our hospital either directly or transferred from other institutions. To ensure diagnostic accuracy, the etiology of acute pancreatitis was systematically evaluated in all patients before confirming the diagnosis of HLAP. Biliary pancreatitis was excluded based on abdominal ultrasonography and/or contrast-enhanced computed tomography (CT), supplemented by magnetic resonance cholangiopancreatography (MRCP) or endoscopic ultrasonography when clinically indicated, together with the absence of gallstones, common bile duct stones, or biliary obstruction. Alcohol-induced pancreatitis was excluded through detailed review of documented alcohol consumption history, drug-induced pancreatitis through comprehensive medication history, and hypercalcemic pancreatitis through admission serum calcium measurements. For transferred patients, complete referral documentation, including outside hospital medical records, laboratory findings, imaging studies, medication history, and discharge summaries, was systematically reviewed through the integrated electronic medical record system and referral documents to confirm the underlying etiology and disease course. The diagnosis of IPN prior to admission was determined based on these records. Patients with pre-existing IPN documented before admission were excluded to avoid misclassification of disease course timing. Patients who developed IPN during the index hospitalization at our center were included and classified into the IPN group according to the predefined outcome criteria described below. The final diagnosis of HLAP was independently confirmed by two experienced gastroenterologists. Any discrepancies were resolved by consensus.

### Data collection

The baseline clinical data and laboratory test results of the patients were retrospectively collected through the electronic medical record system. All laboratory indicators were obtained from the first venous blood sample collected within 24 h after admission. The core exposure variable was the peak plasma D-dimer value measured within 24 h after admission (unit: mg/L). Other variables collected included: age, gender, body mass index (BMI), history of hypertension, and history of diabetes; as well as triglycerides (TG), serum calcium (Ca^2+^), C-reactive protein (CRP), procalcitonin (PCT), albumin (ALB), fibrinogen (FIB), creatinine (Cr), and blood urea nitrogen (BUN) were obtained from the first venous blood sample collected within 24 h of admission. Persistent organ failure (POF) is defined as having a score of ≥ 2 points for any organ (respiratory, circulatory, or renal) based on the modified Marshall scoring system, and this score persists for more than 48 h (18). Therefore, POF could only be ascertained after at least 48 h of clinical observation. The Bedside Index for Severity in Acute Pancreatitis (BISAP) score was calculated within 24 h of admission and includes five indicators: blood urea nitrogen, mental status, systemic inflammatory response syndrome, age, and pleural effusion (19). The BISAP score ranges from 0 to 5, with a higher score indicating a more severe condition. The modified CT severity index (MCTSI) score was calculated based on the abdominal enhanced CT examination completed within 72–96 h after the onset of the disease. Accordingly, MCTSI was not available at admission but became available during the early hospitalization period. This score integrates the degree of pancreatic inflammatory response (0–4 points), the extent of pancreatic necrosis (0–4 points), and extrapancreatic complications (0–2 points), with a total score ranging from 0 to 10 (20). A higher MCTSI score indicates a larger pancreatic necrosis area and more severe extrapancreatic complications. In addition, clinical variables such as pleural effusion, mechanical ventilation, and early enteral nutrition (EEN, initiated within 48 h of onset) were also collected. The earliest time point at which all predictors included in the final model were available was approximately 72–96 h after disease onset. Consequently, the model should be interpreted as an early in-hospital risk stratification tool rather than a purely admission-based prediction model.

### Outcome indicator measurement

The primary outcome measure of this study was infectious pancreatic necrosis. The diagnosis of IPN was based on one of the following criteria: (1) Positive bacterial culture from the puncture and drainage fluid of pancreatic or peripancreatic necrotic tissue; (2) Abdominal enhanced CT scan showing typical “bubble sign” in pancreatic or peripancreatic tissues. All IPN diagnoses were independently confirmed by two radiologists or pancreatic surgeons with senior qualifications, and consensus was reached through consultation when there was disagreement. The diagnosis of IPN referred to the recommended standards in the clinical practice update guideline published by Baron et al. (21) in Gastroenterology. Because pancreatic necrosis may not be apparent on admission, all patients underwent serial clinical and imaging evaluation during hospitalization. Contrast-enhanced CT was performed according to clinical indications, and pancreatic necrosis was diagnosed based on the Revised Atlanta Classification. Patients were subsequently classified as having IPN when predefined diagnostic criteria were fulfilled during follow-up.

### Temporal validity and prevention of information leakage

To ensure that the early in-hospital risk stratification model genuinely forecasts the subsequent development of IPN rather than merely reflecting established disease, we rigorously preserved the temporal sequence between predictor measurement and outcome occurrence. In this study, all predictor variables were derived from data collected during the early phase of hospitalization: Peak D-dimer levels were determined from serial measurements within 24 h of admission, while PCT was measured from the first venous blood sample obtained during the same time window; POF was defined as organ dysfunction persisting for more than 48 h and was thus ascertained after the initial 48-h observation period; and MCTSI scores were calculated based on contrast-enhanced CT examinations performed at 72–96 h after disease onset. The diagnosis of IPN, by contrast, required either positive microbiological cultures from pancreatic necrotic tissue or typical radiological signs of gas collection (the “bubble sign”) on follow-up imaging, and in all cases was established after the completion of the above assessments. Specifically, we reviewed the electronic medical records of all included patients to confirm that the date of IPN diagnosis uniformly postdated both the 48-h POF determination and the 72–96-h CT imaging window. This chronological ordering ensures that our model operates as a genuine early predictor of IPN, without information leakage from the outcome status influencing the predictor variables. Patients in whom IPN could not be definitively ruled out due to insufficient observation time were excluded by the study criteria, as detailed in the inclusion/exclusion section.

### Statistical analysis

All statistical analyses were performed using SPSS 25.0 software (IBM Corp., Armonk, NY, United States) and R software (version 4.5.0, R Foundation for Statistical Computing, Vienna, Austria). For continuous variables, the Shapiro–Wilk normality test was first conducted. Variables that met the normal distribution criteria were expressed as mean ± standard deviation (SD), and comparisons between groups were performed using the independent sample *t*-test. Variables that did not meet the normal distribution criteria were expressed as median (interquartile range), and comparisons between groups were conducted using the Mann–Whitney U test. For categorical variables, the number of cases (percentage) was used for presentation, and comparisons between groups were performed using the χ^2^ test or Fisher’s exact probability method. We fitted a restricted cubic spline (RCS) function to investigate the dose–response relationship between D-dimer levels and IPN risk. Considering that there are very few numbers of cases of IPN in this study, to avoid overfitting and obtain a stable model, a threshold of *p* < 0.05 was decided for variable selection in the univariate logistic regression. However, we acknowledge that relying solely on statistical significance for variable selection may omit clinically important confounders. Therefore, in addition to the variables with *p* < 0.05 in univariate analysis, we also forced age, gender, BMI into the multivariate model as clinically relevant covariates, regardless of their univariate significance. The forced entry technique was applied to yield the predictive model and the calculation of odds ratio (OR) and its 95% confidence interval (CI) was done for various independent risk factors. The predicted probability from this multivariate model was calculated using the logistic regression equation: Logit(P) = *β*₀ + β₁X₁ + β₂X₂ + . + β_k_X_k_, where Xᵢ represents each predictor and βᵢ its corresponding regression coefficient. This predicted probability was used as the “combined model” for subsequent ROC and DCA analyses. By means of the receiver operating characteristic (ROC) curve, we estimate the predictive effectiveness of each indicator alone and in combination for IPN. The area under the curve (AUC), sensitivity, specificity, and the optimal cut-off value were calculated (based on maximizing the Youden index). For comparison of AUCs between different predictive models, the Delong test was performed using the nonparametric approach implemented in SPSS (version 25.0, IBM Corp.). The standard errors and 95% confidence intervals for AUC differences were calculated under the nonparametric assumption as provided by the software output. The Hosmer-Lemeshow goodness-of-fit test, along with calibration curves generated via the bootstrap method using the rms package in R (version 4.5.0), was used to evaluate the calibration of the multivariate logistic regression model. Briefly, 1,000 bootstrap resamples were drawn with replacement from the original dataset; in each iteration, the model was refit and the calibration slope and intercept were calculated, which were then averaged to derive optimism-corrected calibration statistics. The calibration curves were plotted with locally weighted scatterplot smoothing (LOWESS) to visualize the agreement between predicted probabilities and observed outcomes. If *p* > 0.05, it indicates that the consistency between the predicted probability and the actual observed probability is good, meaning that the model calibration is acceptable. Further, clinical decision curve analysis (DCA) was employed to assess the clinical utility of the prediction model using the rmda package in R (version 4.5.0). The net benefit was calculated across a range of threshold probabilities and plotted against the “treat-all” and “treat-none” strategies to evaluate whether the model adds clinical value in guiding decision-making. For the temporal validation cohort, the same logistic regression equation derived from the development cohort was applied to calculate the predicted probability of IPN for each patient. The optimal probability cut-off (determined by the Youden index in the development cohort, based on the combined model’s predicted probability) was then applied to the validation cohort to calculate the corresponding sensitivity, specificity, and other performance metrics. This approach ensures consistency in model evaluation across cohorts and reflects the real-world scenario in which a fixed threshold would be applied prospectively. All tests were two-sided, and a *p*-value of <0.05 was considered statistically significant.

## Results

### Baseline characteristics of patients

A total of 286 HLAP patients were included in this study, among which 47 cases (16.43%) were in the IPN group and 239 cases (83.57%) were in the non-IPN group. There were no statistically significant differences between the IPN group and the non-IPN group in terms of age, gender, BMI, history of hypertension, and history of diabetes (*p* > 0.05) (Table 1). Compared with patients without IPN, patients in the IPN group had significantly higher peak D-dimer levels within 24 h of admission (4.37 ± 2.03 mg/L vs. 2.93 ± 1.59 mg/L, *p* < 0.001), PCT levels (3.90 ± 2.00 ng/mL vs. 2.51 ± 1.69 ng/mL, *p* < 0.001), and MCTSI scores (6.23 ± 1.82 vs. 5.38 ± 2.13, *p* = 0.011), and a higher incidence of POF (29.79% vs. 12.97%, *p* = 0.004). No significant differences were found among other clinical indicators.

**Table 1:** Baseline characteristics of study patients stratified by IPN status.

Variables	Total (*n* = 286)	Non-IPN (*n* = 239)	IPN (*n* = 47)	Statistic	*P*
Age, years, Mean ± SD	49.37 ± 17.26	49.41 ± 16.90	49.19 ± 19.19	t = 0.08	0.937
Male, *n* (%)	200 (69.93)	164 (68.62)	36 (76.60)	χ^2^ = 1.19	0.276
BMI, kg/m^2^, Mean ± SD	27.86 ± 2.38	27.97 ± 2.32	27.34 ± 2.61	t = 1.64	0.101
Hypertension, *n* (%)	80 (27.97)	69 (28.87)	11 (23.40)	χ^2^ = 0.58	0.445
Diabetes mellitus, *n* (%)	54 (18.88)	49 (20.50)	5 (10.64)	χ^2^ = 2.49	0.114
Hospital stay, days, Mean ± SD	9.66 ± 8.18	9.66 ± 8.34	9.64 ± 7.41	t = 0.02	0.986
D-dimer, mg/L, Mean ± SD	3.17 ± 1.75	2.93 ± 1.59	4.37 ± 2.03	t = −4.59	<0.001
TG, mmol/L, Mean ± SD	7.41 ± 2.19	7.35 ± 2.17	7.68 ± 2.25	t = −0.92	0.360
Ca^2+^, mmol/L, Mean ± SD	2.10 ± 0.35	2.11 ± 0.34	2.04 ± 0.37	t = 1.38	0.167
CRP, mg/L, Mean ± SD	227.54 ± 74.38	224.17 ± 70.76	244.69 ± 89.50	t = −1.74	0.084
PCT, ng/mL, Mean ± SD	2.74 ± 1.82	2.51 ± 1.69	3.90 ± 2.00	t = −4.96	<0.001
ALB, g/L, Mean ± SD	30.21 ± 3.84	30.28 ± 3.95	29.82 ± 3.21	t = 0.75	0.454
FIB, g/L, Mean ± SD	7.24 ± 2.29	7.20 ± 2.28	7.42 ± 2.33	t = −0.58	0.566
Cr, μmol/L, Mean ± SD	77.38 ± 24.96	76.36 ± 25.36	82.58 ± 22.36	t = −1.57	0.118
BUN, mmol/L, Mean ± SD	7.18 ± 1.90	7.09 ± 1.91	7.62 ± 1.82	t = −1.76	0.079
MCTSI score, Mean ± SD	5.52 ± 2.11	5.38 ± 2.13	6.23 ± 1.82	t = −2.58	0.011
BISAP score, Mean ± SD	3.03 ± 1.13	2.97 ± 1.15	3.30 ± 1.00	t = −1.80	0.073
POF, *n* (%)	45 (15.73)	31 (12.97)	14 (29.79)	χ^2^ = 8.38	0.004
Pleural effusion, *n* (%)	105 (36.71)	83 (34.73)	22 (46.81)	χ^2^ = 2.47	0.116
Mechanical ventilation, *n* (%)	111 (38.81)	90 (37.66)	21 (44.68)	χ^2^ = 0.82	0.366
EEN, *n* (%)	192 (67.13)	165 (69.04)	27 (57.45)	χ^2^ = 2.39	0.122

IPN, infected pancreatic necrosis; BMI, body mass index; TG, triglycerides; Ca^2+^, serum calcium; CRP, C-reactive protein; PCT, procalcitonin; ALB, Albumin; FIB, fibrinogen; Cr, creatinine; BUN, blood urea nitrogen; MCTSI, modified CT severity index; BISAP, bedside index for severity in acute pancreatitis; POF, persistent organ failure; EEN, early enteral nutrition. *p* values were calculated using independent *t*-test for continuous variables and Chi-square test for categorical variables.

### Non-linear association between D-dimer and IPN risk

**Figure 1:**
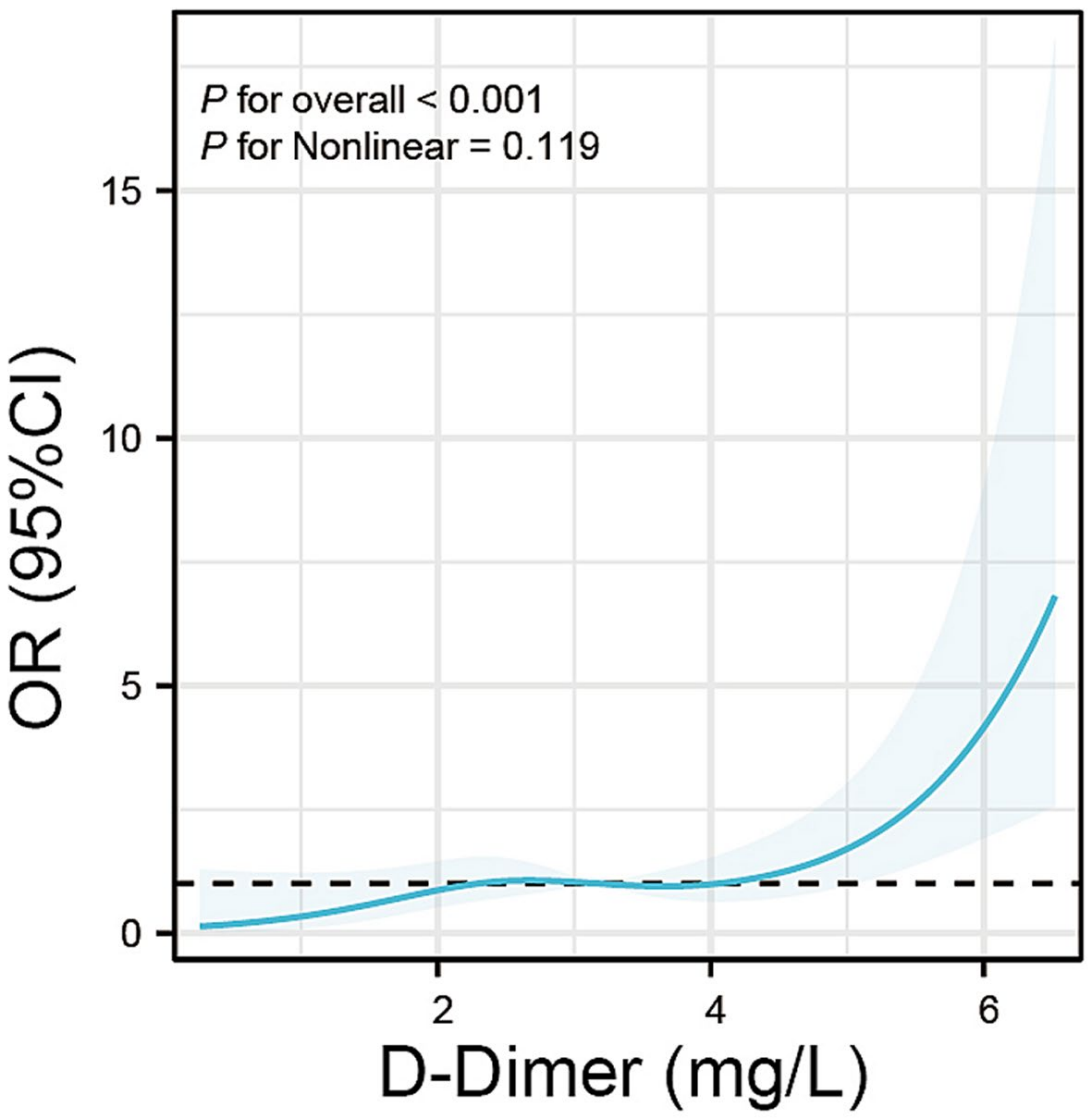
Dose–response relationship between peak 24-h D-dimer levels and risk of IPN.

An RCS model was used to evaluate the dose–response relationship between peak 24-h D-dimer levels and the risk of IPN. As shown in Figure 1, D-dimer levels demonstrated a significant positive association with IPN risk (P for overall < 0.001). The test for non-linearity yielded a *p* value of 0.119, indicating a linear dose–response relationship. The risk of IPN progressively increased with rising D-dimer levels.

### Univariate logistic regression analysis of factors associated with IPN

**Figure 2:**
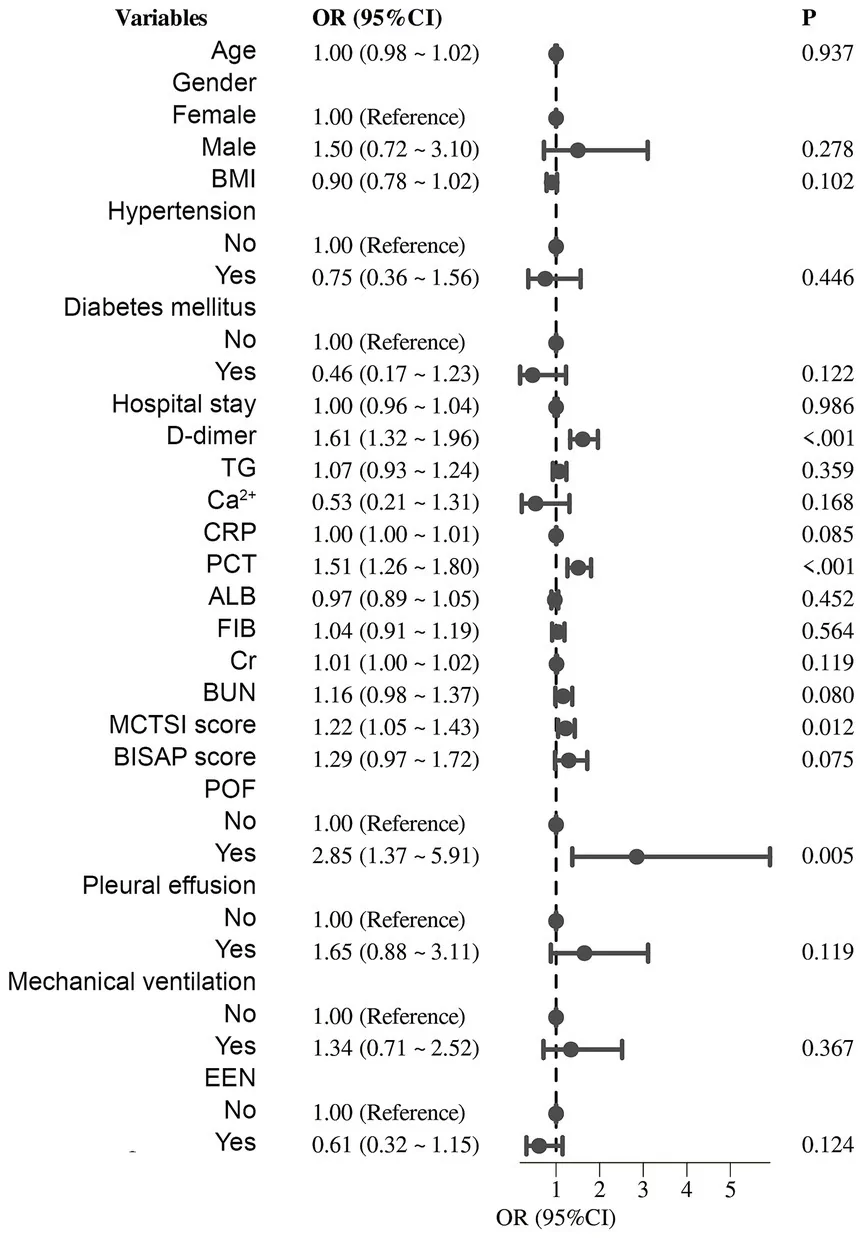
Forest plot of univariate logistic regression analysis for factors associated with IPN.

Figure 2 presents the forest plot of the univariate logistic regression analysis. The results revealed that D-dimer (OR = 1.61, 95% CI: 1.32–1.96, *p* < 0.001), PCT (OR = 1.51, 95% CI: 1.26–1.80, *p* < 0.001), MCTSI score (OR = 1.22, 95% CI: 1.05–1.43, *p* = 0.012), and POF (OR = 2.85, 95% CI: 1.37–5.91, *p* = 0.005) were significantly associated with the risk of IPN. No significant association was found between other factors and the risk of IPN (all *p* > 0.05).

### Multivariate logistic regression analysis of independent risk factors for IPN

Variables with *p* < 0.05 in univariate analysis (D-dimer, PCT, MCTSI score, and POF) were entered into a multivariate logistic regression model using the enter method (Model 1). To address the concern that relying solely on statistical significance for variable selection may omit clinically important confounders, we additionally forced age, sex, and BMI into the model regardless of their univariate significance (Model 2). As shown in Table 2, D-dimer (per 1 mg/L increase: OR = 1.67, 95% CI: 1.34–2.08, *p* < 0.001), PCT (per 1 ng/mL increase: OR = 1.57, 95% CI: 1.28–1.92, *p* < 0.001), MCTSI score (per 1 point increase: OR = 1.25, 95% CI: 1.05–1.50, *p* = 0.014), and POF (OR = 3.11, 95% CI: 1.28–7.56, *p* = 0.012) were independent risk factors for IPN in patients with HLAP in Model 1. In Model 2, after additional adjustment for age, sex, and BMI, the results remained essentially unchanged (D-dimer: OR = 1.76, 95% CI: 1.39–2.24, *p* < 0.001; PCT: OR = 1.62, 95% CI: 1.31–2.01, *p* < 0.001; MCTSI: OR = 1.30, 95% CI: 1.08–1.57, *p* = 0.006; POF: OR = 3.39, 95% CI: 1.36–8.44, *p* = 0.009), indicating that the associations were robust to adjustment for these clinically relevant covariates.

**Table 2:** Multivariate logistic regression analysis of independent risk factors for IPN.

Variables	Model 1			Model 2		
	OR	95% CI	*P*	OR	95% CI	*P*
D-dimer, per 1 mg/L increase	1.67	1.34 ~ 2.08	<0.001	1.76	1.39 ~ 2.24	<0.001
With POF	3.11	1.28 ~ 7.56	0.012	3.39	1.36 ~ 8.44	0.009
PCT, per 1 ng/mL increase	1.57	1.28 ~ 1.92	<0.001	1.62	1.31 ~ 2.01	<0.001
MCTSI score, per 1 point increase	1.25	1.05 ~ 1.50	0.014	1.30	1.08 ~ 1.57	0.006

IPN, infected pancreatic necrosis; OR, odds ratio; CI, confidence interval; POF, persistent organ failure; PCT, procalcitonin; MCTSI, modified CT severity index. Model 1 included D-dimer, PCT, MCTSI score, and POF as independent variables (forced entry). Model 2 was additionally adjusted for age, sex, and BMI as clinically relevant covariates in a sensitivity analysis.

### Construction of a nomogram for predicting IPN risk

**Figure 3:**
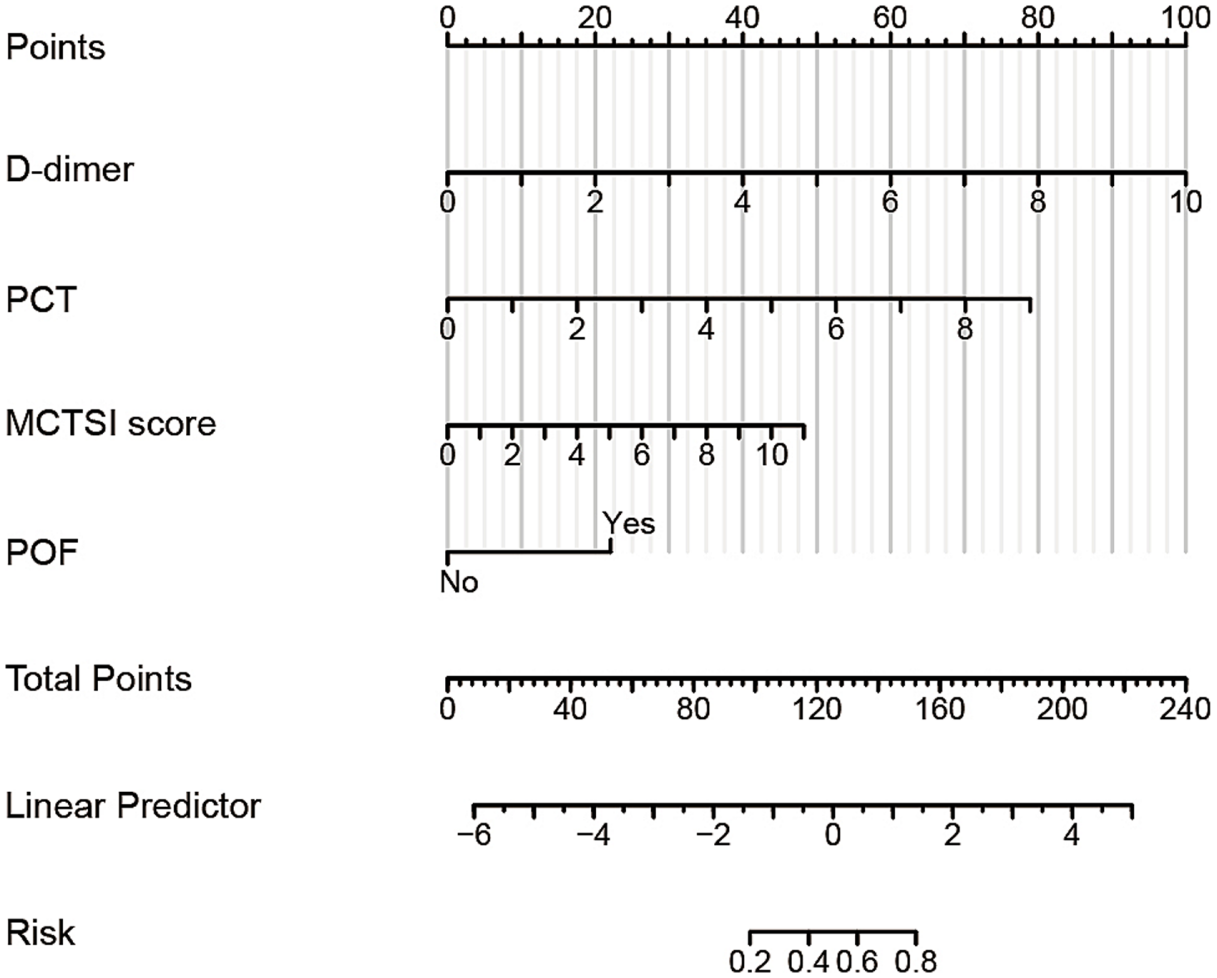
Nomogram for predicting the risk of IPN in patients with HLAP.

Based on the four independent risk factors identified through multivariate Logistic regression analysis (D-dimer, PCT, MCTSI score, and POF), a nomogram model for predicting the risk of secondary IPN in patients with HLAP was constructed (Figure 3). The predicted probability of IPN was calculated using the following logistic regression equation: Logit(P) = −6.44 + 0.51 × (D-dimer, mg/L) + 0.45 × (PCT, ng/mL) + 0.23 × (MCTSI score) + 1.13 × (POF, where 1 = present and 0 = absent). This equation represents the combined model, in which each predictor contributes according to its respective regression coefficient (*β*), thereby providing a weighted risk estimate rather than a simple unweighted summation of the four variables. This nomogram calculates the total score by adding the corresponding scores of each variable, and then maps out the predicted probability of IPN, enabling a visual and individualized assessment of the IPN risk. The predictive performance of individual indicators and the combined model is summarized in Table 3. At the optimal cutoff value (determined by the Youden index), the combined model demonstrated an accuracy of 0.80, sensitivity of 0.70, specificity of 0.82, positive predictive value of 0.43, and negative predictive value of 0.93. Notably, the combined model exhibited superior sensitivity and specificity compared to any single indicator, indicating its excellent discriminative ability for IPN.

**Table 3:** Predictive performance of individual biomarkers and combined model for IPN.

Variable	AUC (95%CI)	Accuracy	Sensitivity	Specificity	PPV	NPV	Cut off
D-dimer (mg/L)	0.70 (0.62–0.79)	0.79	0.47	0.85	0.38	0.89	4.605
POF	0.58 (0.51–0.65)	0.78	0.30	0.87	0.31	0.86	0.224
PCT (ng/mL)	0.69 (0.61–0.77)	0.53	0.87	0.46	0.24	0.95	2.05
MCTSI score	0.62 (0.54–0.70)	0.54	0.70	0.51	0.22	0.90	5.5
Combined model	0.82 (0.75–0.89)	0.80	0.70	0.82	0.43	0.93	–

IPN, infected pancreatic necrosis; AUC, area under the receiver operating characteristic curve; CI, confidence interval; PPV, positive predictive value; NPV, negative predictive value; POF, persistent organ failure; PCT, procalcitonin; MCTSI, modified CT severity index. The “Combined model” represents the predicted probability derived from the multivariate logistic regression equation incorporating D-dimer, PCT, MCTSI score, and POF as weighted variables based on their respective regression coefficients.

### Discrimination, calibration, and clinical utility of the predictive model

**Figure 4:**
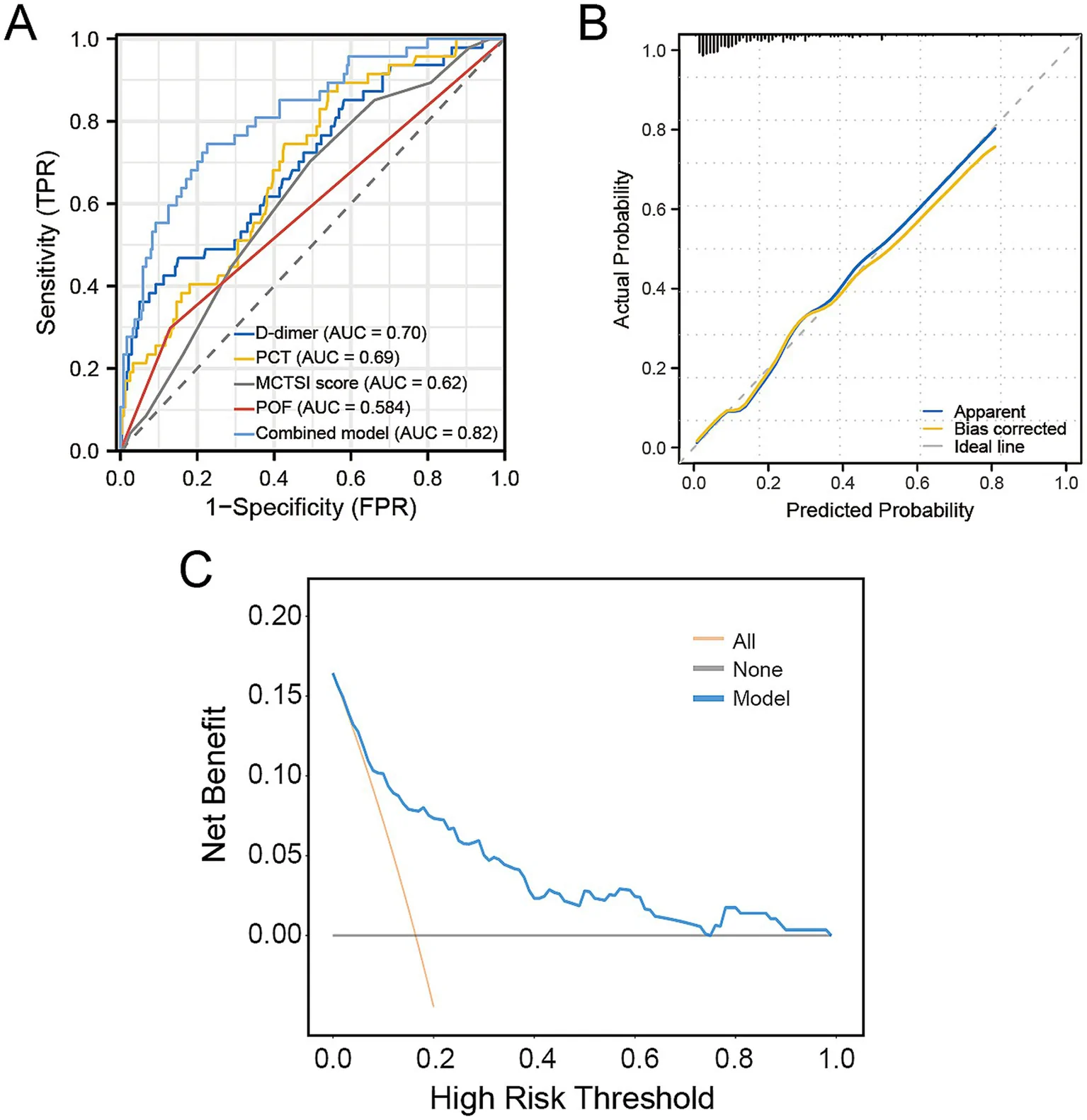
Performance of the combined predictive model in the derivation cohort. **(A)** ROC curves for D-dimer, POF, PCT, MCTSI score, and the combined model. **(B)** Calibration curve of the combined model. **(C)** DCA for the combined model.

ROC analysis showed that the AUCs for D-dimer, POF, PCT, and the MCTSI score as independent predictors of IPN were 0.70 (95% CI: 0.62–0.79), 0.58 (95% CI: 0.51–0.65), 0.69 (95% CI: 0.61–0.77), and 0.62 (95% CI: 0.54–0.70), respectively (Figure 4A). The AUC of the combined predictive model using all four indicators —defined as the predicted probability derived from the logistic regression equation above— was 0.82 (95% CI: 0.75–0.89). The calibration curve (Figure 4B) showed good agreement between the predicted probabilities and the observed probabilities in the combined predictive model, with a *p*-value of 0.806 for the Hosmer-Lemeshow test. DCA revealed that the combined model provided a higher net benefit than the “treat-all” and “treat-none” strategies across a threshold probability range of 0.1 to 0.7, demonstrating favorable clinical utility (Figure 4C).

### Comparison of AUCs among different predictive models

Delong’s test was performed to compare the AUCs of different predictive models (Table 4). The combined model had a significantly higher AUC than each of the four individual predictors (D-dimer, PCT, MCTSI score, and POF; all *p* < 0.05). Furthermore, the AUCs of D-dimer and PCT were both significantly higher than that of POF (both *p* < 0.05). No significant differences in AUC were observed between D-dimer and PCT, nor between MCTSI score and other individual predictors (all *p* > 0.05).

**Table 4:** Pairwise comparisons of AUCs among different predictive models using Delong’s test.

Comparison	AUC_1_	AUC_2_	Difference	SE	95% CI	*P*
Combined model vs. D-dimer	0.82	0.70	0.116	0.276	0.043–0.189	0.002
Combined model vs. PCT	0.82	0.69	0.127	0.270	0.057–0.197	<0.001
Combined model vs. MCTSI	0.82	0.62	0.196	0.275	0.099–0.293	<0.001
Combined model vs. POF	0.82	0.58	0.234	0.263	0.153–0.314	<0.001
D-dimer vs. PCT	0.70	0.69	0.011	0.289	−0.130–0.109	0.862
D-dimer vs. MCTSI	0.70	0.62	0.079	0.292	−0.039–0.198	0.189
D-dimer vs. POF	0.70	0.58	0.117	0.282	0.001–0.234	0.048
PCT vs. MCTSI	0.69	0.62	0.069	0.287	−0.056–0.194	0.279
PCT vs. POF	0.69	0.58	0.107	0.273	0.015–0.199	0.023
MCTSI vs. POF	0.62	0.58	0.038	0.278	−0.067–0.143	0.478

AUC, area under the receiver operating characteristic curve; SE, standard error; CI, confidence interval; PCT, procalcitonin; POF, persistent organ failure; MCTSI, modified CT severity index. *P* values and SE were calculated using Delong’s test for paired AUC comparisons as implemented in SPSS version 25.0 (nonparametric assumption). The 95% CIs for AUC differences were derived directly from the SPSS output.

### Performance of the predictive model in the clinical validation cohort

**Figure 5:**
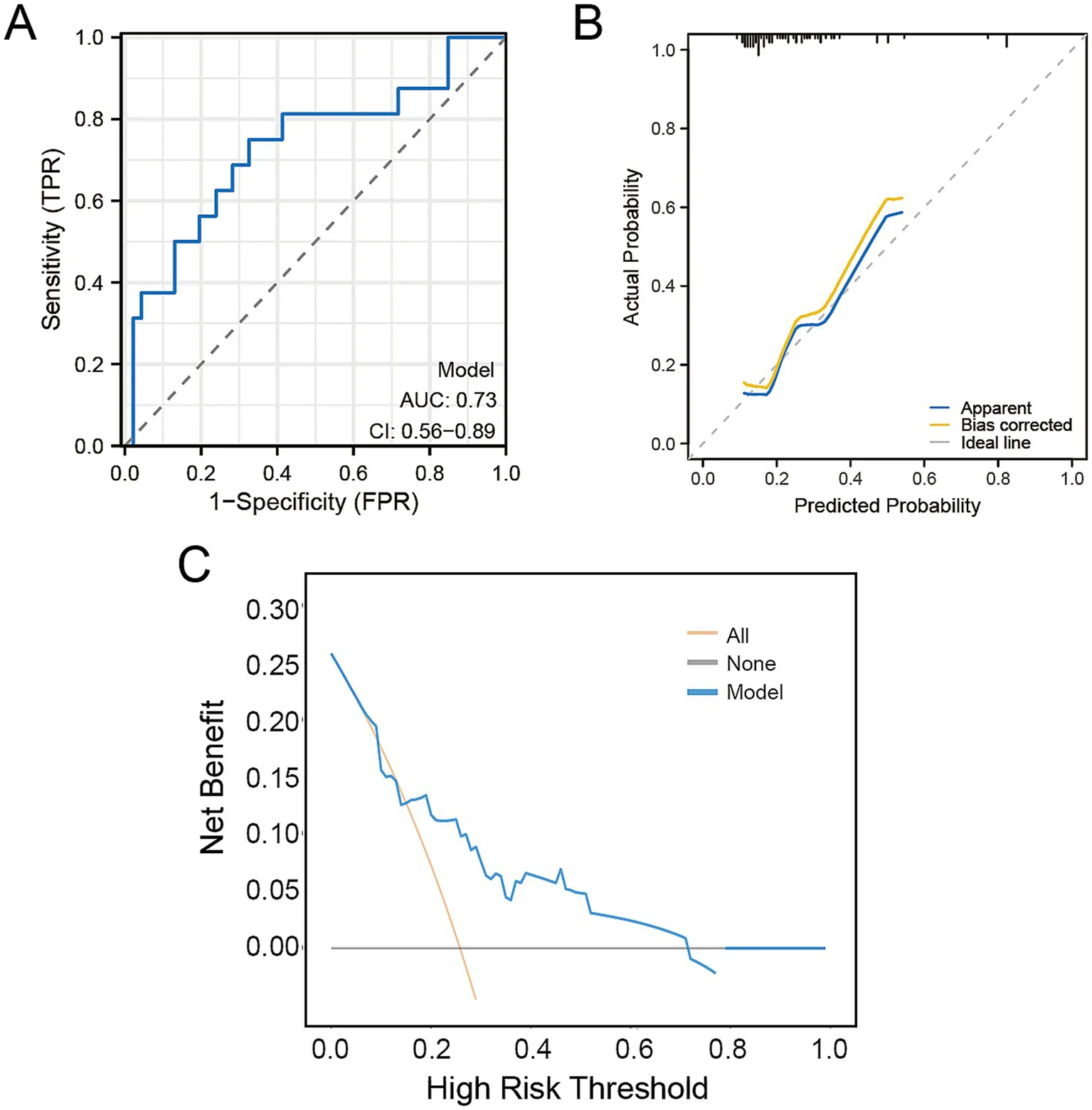
Performance of the combined predictive model in the temporal validation cohort. **(A)** ROC curve of the combined model in the validation cohort. **(B)** Calibration curve of the combined model in the validation cohort. **(C)** DCA for the combined model in the validation cohort.

In an independent clinical validation cohort comprising 62 HLAP patients, the combined model achieved an AUC of 0.73 (95% CI: 0.56–0.89) for predicting IPN (Figure 5A), corresponding to a reduction of 0.09 compared with the derivation cohort AUC of 0.82. Delong’s test showed no statistically significant difference between the two AUCs (*p* = 0.319). However, the wide 95% confidence interval (0.56–0.89) reflects the limited sample size of the validation cohort (*n* = 62, with only 16 IPN events), and the observed AUC reduction should be interpreted cautiously. This temporal validation demonstrates that the model retains moderate discriminative ability within the same institution over a different time period, but it does not constitute true external validation across different centers or populations. Confusion matrix analysis was performed using the probability cut-off derived from the development cohort (based on the Youden index). Under this fixed threshold, the model achieved an accuracy of 0.69, sensitivity of 0.75, specificity of 0.67, PPV of 0.44, and NPV of 0.89 (Table 5). The 95% confidence interval for the validation AUC was relatively wide (0.56–0.89), reflecting the limited sample size of the validation cohort (*n* = 62, with only 16 IPN events). The calibration curve demonstrated good agreement between predicted and observed probabilities (Hosmer-Lemeshow *p* = 0.697) (Figure 5B). DCA showed that the model line remained above the “treat-all” and “treat-none” lines across a threshold probability range of 0.2 to 0.7 (Figure 5C).

**Table 5:** Predictive performance of the combined model in the clinical validation cohort.

IPN	Predicted Non-IPN	Predicted IPN	Total
Actual Non-IPN	31	15	46
Actual IPN	4	12	16
Total	35	27	62

Performance metrics: Accuracy = 0.69, Sensitivity = 0.75, Specificity = 0.67, PPV = 0.44, NPV = 0.89. IPN, infected pancreatic necrosis; PPV, positive predictive value; NPV, negative predictive value. Validation cohort consisted of 62 independent patients from the same institution.

## Discussion

This retrospective study included 286 patients with HLAP and systematically investigated the association between D-dimer levels within 24 h of admission and secondary IPN. The findings indicate that D-dimer is an independent risk factor for the development of IPN in patients with HLAP, and that there is a linear dose–response relationship between D-dimer levels and risk. Furthermore, PCT, the MCTSI score and POF were also identified as independent predictors. The early in-hospital risk stratification model constructed using the four aforementioned indicators demonstrated significantly superior discriminatory power (AUC = 0.82), calibration and clinical net benefit compared to any single indicator, and demonstrated moderate generalizability in an independent temporal validation cohort, though the observed reduction in AUC and the relatively wide confidence interval indicate that further external validation in larger cohorts is warranted. This finding provides new evidence-based support for the early risk stratification and personalized prevention and treatment of IPN in patients with HLAP. It should be noted that the combined model was not intended to function as a purely admission-based prediction tool. Although D-dimer was measured within 24 h of admission, the combined model also incorporated persistent organ failure and MCTSI, both of which become available during the early course of hospitalization. Therefore, this model is more appropriately interpreted as an early in-hospital risk stratification tool, integrating admission laboratory findings with dynamic clinical and imaging information obtained during the first several days after admission. In addition, given the retrospective observational design of this study, these findings should be interpreted as associations rather than causal relationships, and residual confounding from unmeasured factors cannot be excluded.

D-dimer, as a specific degradation product of cross-linked fibrin, is a sensitive indicator of the activation status of the body’s coagulation and fibrinolytic systems (22). In recent years, the value of D-dimer in assessing the severity of acute pancreatitis has received increasing attention. Studies conducted in mixed-etiology acute pancreatitis populations have shown that elevated D-dimer levels are associated with more severe disease courses, higher rates of organ failure, and increased mortality (10, 11, 23). It is important to note, however, that these studies enrolled patients with acute pancreatitis of various etiologies and used overall disease severity—rather than infected pancreatic necrosis specifically—as their primary endpoint. In contrast, the present study focuses specifically on the HLAP population and on IPN as the outcome of interest, thereby addressing a more targeted clinical question. Within the HLAP-specific literature, our findings are consistent with several recent reports. Gou et al. (24) demonstrated that D-dimer had an AUC of 0.753 for predicting severe HLAP and was an independent risk factor for disease severity. Cao et al. (25) confirmed D-dimer as an independent predictor for hypertriglyceridaemic severe acute pancreatitis. Zhang et al. (26) showed that elevated D-dimer levels were significantly associated with severe hyperlipidemic acute pancreatitis, with their predictive model achieving an AUC of 0.834. Similarly, Li et al. (27) identified elevated D-dimer as an independent risk factor for disease progression in HTG-AP patients across different age groups, with AUCs of 0.653 and 0.741 in young and middle-aged/elderly patients, respectively. Together, these studies corroborate one another and reinforce the central role of D-dimer in assessing disease severity and progression in hypertriglyceridaemic acute pancreatitis. Our study extends these observations by specifically linking D-dimer to the subsequent development of IPN, a clinically more consequential endpoint than overall severity classification.

From the perspective of pathophysiological mechanisms, the association between elevated D-dimer levels in patients with HLAP and increased risk of IPN may involve multiple links. Firstly, in the state of hypertriglyceridemia, the serum triglyceride level is closely related to the severity of the disease. Especially within 48 h after the onset of the disease, the higher the triglyceride level, the greater the risk of pancreatic necrosis and organ failure (28). High levels of free fatty acids can directly damage the vascular endothelium, activate the extrinsic coagulation pathway, and lead to microthrombosis and impaired pancreatic microcirculation (29). Microcirculation disorders not only aggravate the ischemic necrosis of pancreatic tissue, but also can prevent antibiotics from penetrating into the necrotic tissue through the “no-reflow” phenomenon, creating conditions for bacterial colonization and reproduction. Secondly, once the coagulation system is activated, thrombin binds to protease-activated receptors on the surface of platelets and immune cells, inducing the release of inflammatory cytokines and forming a “coagulation-inflammation” positive feedback loop (30). This interaction between coagulation and inflammation may amplify the systemic inflammatory response, exacerbate damage to the intestinal barrier function, and promote bacterial translocation—which is considered the primary source of infection in IPN (31). Furthermore, elevated D-dimer levels may in themselves reflect extensive necrotic tissue and a heavy local inflammatory burden. A study by Husu et al. (8) found that the anatomical extent of pancreatic necrosis is an independent risk factor for IPN, and that extensive necrosis is often associated with more pronounced coagulation disorders. Consequently, D-dimer is not only a marker of activation of the coagulation/fibrinolytic system, but is also likely to provide a comprehensive reflection of the extent of pancreatic necrosis, the severity of microcirculatory dysfunction, and the systemic inflammatory burden; this explains its high predictive value for IPN. It is worth noting that the ROC analysis in this study revealed a linear relationship between D-dimer and the risk of IPN rather than a threshold effect, suggesting that clinically, even mild to moderate elevations in D-dimer levels should raise concern, rather than focusing solely on a specific cut-off value.

In addition to D-dimer, this study also found that PCT, the MCTSI score and POF to be independent risk factors for IPN, which is highly consistent with the existing literature. A meta-analysis by Tarján et al. (32) showed that persistently high levels of PCT and CRP 72 h after admission are good predictors of infective necrosis (with AUCs of 0.86 and 0.88, respectively). Zhu et al. (33) also confirmed that the combination of PCT, the neutrophil/lymphocyte ratio, and the MCTSI score could achieve an AUC of 0.92 for predicting IPN. In a broader context of AP severity prediction, Salehi et al. (34) recently evaluated the diagnostic value of the neutrophil-to-lymphocyte ratio (NLR), platelet-to-lymphocyte ratio (PLR), and systemic immune-inflammation index (SII) in a cohort of 112 AP patients using the MCTSI as the reference standard. Their multivariate model incorporating all three ratios achieved an AUC of approximately 0.74, with PLR demonstrating the highest Youden index (0.425), NLR showing the best sensitivity (77.78%), and SII exhibiting the highest specificity (84.29%) at their respective optimal cut-offs. These findings align with the heterogeneous landscape of inflammatory marker performance, where different indices may excel in different aspects of risk stratification. Notably, our study complements these observations by focusing on a more specific outcome (IPN) and a distinct disease subset (HLAP), highlighting the potential additive value of D-dimer as a coagulation-related marker beyond conventional inflammatory indices. The MCTSI score provides a comprehensive assessment of the extent of pancreatic necrosis and extrapancreatic complications; in this study, the MCTSI score in the IPN group was significantly higher than that in the non-IPN group. This finding is consistent with the results of Cao et al. (25), which indicated that the AUC of the MCTSI score in predicting HLAP was 0.83. POF was the predictor with the highest OR value in this study (OR = 3.11), reflecting the bidirectional relationship between IPN and organ failure: organ failure is not only an important risk factor for IPN, but also the most serious clinical consequence after the occurrence of IPN (35). Our findings are consistent with those of Husu et al. (8), which demonstrated that persistent organ failure is a common feature in patients with necrotizing SAP, while the ICU stay and total hospital stay of patients with IPN were significantly prolonged. In contrast, no significant difference in total hospital length of stay was observed in the present cohort. This discrepancy may be attributable to the relatively small number of IPN cases and to variations in discharge practices and referral patterns in our institution. In addition, some patients with IPN underwent transfer to specialized centers or continued treatment in local hospitals after initial stabilization, which may have attenuated the observed difference in hospitalization duration. It is noteworthy that in our cohort, CRP levels and BISAP scores showed only trends toward significance in univariate analysis, despite their well-established roles in predicting AP severity. Several factors may explain this. First, both markers were assessed at a single time point (within 24 h of admission), whereas serial measurements may better capture the dynamic inflammatory process leading to IPN. Second, CRP and PCT—both acute-phase reactants—likely share substantial collinearity, and PCT may have outperformed CRP as a more specific marker of bacterial infection in this context. Third, the BISAP score includes age and pleural effusion, neither of which differed significantly between groups, which may have diluted its discriminative power. These findings highlight the importance of disease-specific validation.

The AUC of the early in-hospital risk stratification model constructed in this study reached 0.82, which was significantly better than that of each individual indicator. This is consistent with the research approach of Zhang et al. (36)—that is, integrating multiple dimensions of indicators can significantly improve the prediction efficiency. However, unlike these studies which mainly focus on the overall severity of the disease, this research focuses on the prediction of the specific complication of IPN, and has a more targeted clinical relevance. In the validation cohort, the AUC of the combined model was 0.73; although this was slightly lower than that in the derivation cohort, the D’Long test indicated that the difference between the two was not statistically significant, suggesting that the model possesses acceptable generalization ability. Several practical considerations should be addressed before translating our model into clinical practice. First, the PPV of 0.43 indicates that over half of high-risk alerts would be false positives. We therefore propose the model as a screening trigger for enhanced surveillance rather than a diagnostic tool: high-risk patients could be prioritized for: (1) enhanced surveillance with more frequent vital sign and laboratory monitoring (including daily PCT and CRP trends); (2) scheduled contrast-enhanced CT follow-up for early detection of necrosis progression and extrapancreatic complications; (3) earlier consideration of microbiological testing (blood cultures and, when clinically indicated, fine-needle aspiration) once clinical suspicion of infection arises; and (4) proactive nutritional support and avoidance of unnecessary empirical antibiotics unless infection is confirmed. The model’s utility lies in raising clinical suspicion and allocating limited monitoring resources to those at greatest risk, rather than dictating immediate therapeutic interventions. Second, while the NPV of 0.93 appears excellent, we acknowledge that this is largely driven by the low IPN prevalence (16.4%) in our cohort. In higher-prevalence populations (e.g., ICU settings), the NPV would likely be lower. Nevertheless, the high NPV provides reassurance to avoid unnecessary monitoring in low-risk patients, potentially reducing resource utilization. Third, the DCA threshold should be selected based on clinical context: lower thresholds (e.g., 0.2–0.3) for enhanced surveillance, and higher thresholds (e.g., 0.5–0.7) for invasive diagnostics. The optimal threshold should be tailored to institutional resources and physician risk tolerance, rather than applied as a fixed cutoff. Prospective implementation studies are needed to validate the model’s real-world impact. The present study was designed as an admission-based prediction model intended for early clinical risk stratification. Therefore, the study population included all eligible patients with HLAP rather than only those with established pancreatic necrosis. Restricting the analysis to patients with necrotizing pancreatitis would address a different clinical question—namely, prediction of infection after necrosis has already occurred—rather than the early identification of patients at risk for future IPN.

This study has the following limitations. Firstly, as this is a single-center retrospective study, selection bias and information bias may still be present despite the inclusion of a time-matched cohort. And, the temporal validation cohort was derived from the same institution and comprised only 62 patients, which limits the assessment of model generalizability. True external validation requires independent cohorts from different geographic regions and healthcare settings. The reduction in AUC from 0.82 to 0.73 and the wide confidence interval underscore the need for caution when extrapolating our findings to other populations. Moreover, due to the observational nature of the study, our findings represent statistical associations rather than causal relationships. Despite adjustment for multiple confounders in the multivariate analysis, we cannot exclude the possibility of residual confounding by unmeasured factors, such as genetic predisposition, detailed medication history (e.g., prior anticoagulant or antiplatelet therapy), smoking status, or other lifestyle factors that were not captured in our electronic medical records. Secondly, there may be variations in D-dimer testing methods and normal reference ranges between hospitals; external validation is required to determine whether the cut-off values established in this study are applicable to other centers. Thirdly, no formal sample size calculation based on contemporary prediction-model methodology was performed because of the retrospective design. Although the number of outcome events was considered during model development, we acknowledge that the traditional EPV criterion alone is insufficient to fully assess sample size adequacy. Therefore, larger multicenter cohorts are warranted to further validate the stability and generalizability of the proposed model. Furthermore, we forced age and BMI into the multivariate model as clinically relevant covariates in a sensitivity analysis, and the results remained essentially unchanged. However, the relatively small IPN case number precludes adjustment for additional potential confounders (such as diabetes control status and other comorbidities) beyond the selected predictors. Fourthly, this study did not include certain emerging biomarkers [such as angiopoietin-2 and soluble PD-L1 (37)] for comparative analysis; the value of these markers in the early prediction of IPN requires further exploration. Fifthly, the MCTSI score used in this study relies on contrast-enhanced CT scans performed 72–96 h after onset. In our initial screening, 10 patients (3.2% of the 313 initially identified patients) were excluded from the final analysis specifically because they lacked contrast-enhanced CT data within the required time window. The lack of contrast enhancement may have limited the accuracy of necrosis assessment in these cases. This requirement for contrast-enhanced CT at a specific time window limits the model’s applicability in patients with contraindications to contrast agents, and alternative imaging strategies or biomarkers for this subgroup warrant further investigation. Sixthly, although we emphasize early prediction in this study, it is important to acknowledge that the combined model requires the MCTSI score, which relies on contrast-enhanced CT performed 72–96 h after onset. Thus, the model is more accurately characterized as a tool for early risk stratification during the initial course of hospitalization rather than a prediction model based solely on admission data. To mitigate the risk of information leakage, we excluded any patient in whom IPN was diagnosed before the assessment of all predictors was complete. However, we acknowledge that in real-world clinical practice, the exact timing of IPN onset may be difficult to determine, and some patients may have had subclinical infection before formal diagnosis. Future prospective studies should incorporate serial biomarker measurements and dynamic imaging to better capture the temporal evolution of IPN. This distinction should be considered when interpreting the clinical applicability of our findings. Future studies should conduct multicentre, large-scale prospective trials to externally validate this model and explore the predictive value of combining D-dimer with other inflammatory markers, with a view to further improving the accuracy of early IPN identification. Moreover, the moderate PPV underscores the need for larger cohorts to refine model performance, and prospective implementation studies are warranted to determine optimal threshold probabilities and evaluate whether model-guided surveillance improves clinical outcomes.

## Conclusion

In this retrospective study involving 286 patients with HLAP, researchers found that an elevated D-dimer level measured within 24 h of admission was independently associated with IPN, exhibiting a linear dose–response relationship (OR = 1.67 per 1 mg/L increase). POF, MCTSI score, and procalcitonin were also independent predictors. The early in-hospital risk stratification model that consists of the four indicators displayed good ability to differentiate between those who will go on to get a remote impairment and those who do not, as measured by the AUC of 0.82. Moreover, it showed good calibration and clinical net benefit, and demonstrated moderate generalizability in an independent temporal validation cohort (AUC = 0.73, 95% CI: 0.56–0.89), though the wide confidence interval and reduction in AUC compared with the derivation cohort suggest that the model’s performance should be interpreted with caution until further validated in larger external cohorts. This model may assist in early risk stratification of IPN in HLAP patients and inform clinical decision-making and individualized management. However, given the moderate performance observed in the temporal validation cohort (AUC = 0.73, 95% CI: 0.56–0.89), the single-center retrospective design, and the limited number of IPN events, the model should be considered a preliminary tool that requires further external validation in larger, multicenter prospective cohorts before widespread clinical application.

## Data Availability

The raw data supporting the conclusions of this article will be made available by the authors, without undue reservation.
